# Surgery first – protocols and techniques for orthodontic treatment

**DOI:** 10.1186/1746-160X-10-S1-O2

**Published:** 2014-12-12

**Authors:** Timo Peltomäki

**Affiliations:** 1 Oral and Maxillofacial Unit, Tampere University Hospital, Tampere, Finland

## 

Surgery first concept in orthognathic surgery has been introduced less than a decade ago. The advantages of this approach are claimed to be:

• Shorter entire treatment period.

• Better satisfaction because of the patients’ main complaint, facial aesthetics, is achieved and improved at the beginning of the treatment.

• Postoperative orthodontic tooth movement is accelerated because of favourable metabolic bony changes due to surgery.

Evidence to support these claims is, however, weak. One may further question if surgical stability is compromised without presurgical orthodontics, which in conventional orthognathic surgery is thought to maximize stable postoperative occlusion and reduce relapse tendency. Evident longer postoperative orthodontics treatment duration in the surgery first concept may also cause problems, since it has been reported, that longer than nine months postoperative treatment is no longer well tolerated by the patients.

From the orthodontic point of view reasons for surgery first include:

• Early surgery in patients with severe sleep apnea.

• Deep bite and scissors bite cases where presurgical orthodontic treatment is not possible.

• Class III patients in whom presurgical treatment would lead to significant worsening of the facial outlook.

Surgery first concept requires close collaboration with the surgeon and orthodontist to plan the surgery, since in many cases surgical overcorrection has to be considered to compensate for the space required for final tooth alignment. Postsurgical orthodontic mechanotherapy is also demanding, but modern skeletal anchorage systems offer here a valuable tool.

Without proper scientific evidence surgery first has to be considered as an experimental concept with a need of much further investigation.

